# Coauthors’ Network of Solastalgia. Comment on Galway, L.P.; Beery, T.; Jones-Casey, K.; Tasala, K. Mapping the Solastalgia Literature: A Scoping Review Study. *Int. J. Environ. Res. Public Health* 2019, *16*, 2662.

**DOI:** 10.3390/ijerph17072308

**Published:** 2020-03-30

**Authors:** Alvaro J. Idrovo

**Affiliations:** Public Health Department, School of Medicine, Universidad Industrial de Santander, Carrera 32 #29-32 Bucaramanga 680002, Santander, Colombia; idrovoaj@yahoo.com.mx

Recently, a very interesting article on solastalgia was published in the *International Journal of Environmental Research and Public Health* [1]. In this article the authors review the publications on this topic. Since solastalgia is a new concept in the scientific literature, it is an interesting case to analyze how a neologism is disclosed among scientists. According to Fleck, knowledge is socially constructed through the so-called “collective thinking”, that is, groups of individuals who exchange ideas or maintain intellectual exchange relations. These thought groups enable the expression of the “style of thinking”, which turns out to be a common belief system with elements that are considered obvious, and apply the same method as a form of cognition [2].

For this exploration, we conducted a social network analysis with coauthors [3] of publications reported in the article by Galway et al. First, a symmetric matrix was plotted using the NetDraw 2.168 program [4] and the analyses were performed with UCINET [5]. Figure 1 shows the coauthors network built with data. There were 28 different publications with 58 coauthors and 208 ties. The author with the most publications was Glenn Albrecht with eight publications, followed by Freeman, Connor, and Higginbotham with four publications, and Usher, Mills, and Warsini with three publications. The average degree was 3.586, and the authors with the highest degrees were Albrecht, Connor, Freeman, and Higginbotham. All these authors are in the same network.

The main limitation of this analysis is related to the language of publications included. Galway et al. only included publications in English, thus any another language was excluded. However, it is possible that the inclusion of these publications does not greatly change the coauthors’ network. Results suggest that solastalgia is still a new concept in the scientific literature. Its main exponent is the person who coined the term [6], and the most solid scientific group that works in solastalgia is constituted by his direct collaborators. There are other groups, all dispersed, with fewer members in different regions. The lack of connections between some of the coauthors in the network suggests that the transmission of knowledge on solastalgia has been performed through the dissemination of documents and not through personal contact sharing research activities. This is frequently the case in times when is possible to have access to electronic documents. Without a doubt, solastalgia is and will be a topic of interest in the future linking environmental health, mental health and sustainability.

## Figures and Tables

**Figure 1:**
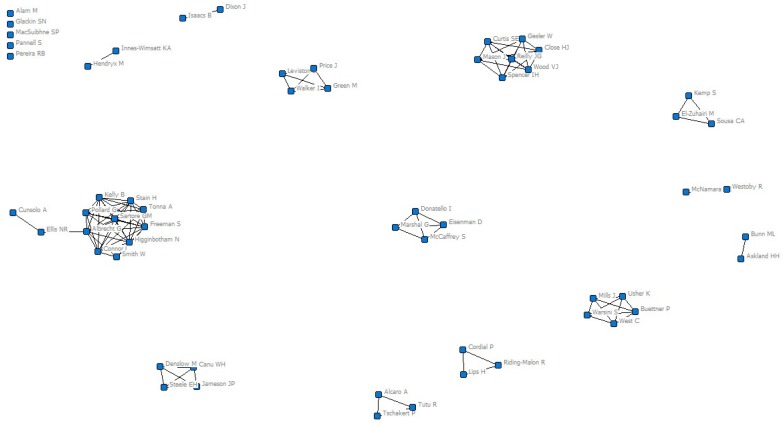
Coauthors’ network of solastalgia.

## References

[1] Galway L.P., Beery T., Jones-Casey K., Tasala K. (2019). Mapping the solastalgia literature: A scoping review study. Int. J. Environ. Res. Public Health.

[2] Fleck L. (1979). The Genesis and Development of a Scientific Fact.

[3] Newman M.E. (2004). Coauthorship networks and patterns of scientific collaboration. Proc. Natl. Acad. Sci. USA.

[4] Borgatti S.P. (2002). NetDraw: Graph Visualization Software.

[5] Borgatti S.P., Everett M.G., Freeman L.C. (2002). Ucinet 6 for Windows: Software for Social Network Analysis.

[6] Albrecht G. (2006). Solastalgia. Environmental damage has made it possible to be homesick without leaving home. Altern. J..

